# Comparison of fifteen SARS-CoV-2 nucleic acid amplification test assays used during the Canadian Laboratory Response Network’s National SARS-CoV-2 Proficiency Program, May 2020 to June 2021

**DOI:** 10.14745/ccdr.v49i05a03

**Published:** 2023-05-01

**Authors:** Charlene Ranadheera, Kym Antonation, Cindi Corbett

**Affiliations:** 1 Health Security and Response Division, National Microbiology Laboratory, Public Health Agency of Canada, Winnipeg, MB

**Keywords:** PCR, SARS-CoV-2, nucleic acid amplification test, COVID-19

## Abstract

**Background:**

On March 11, 2020, the World Health Organization declared a pandemic caused by the recently emerged severe acute respiratory syndrome coronavirus 2 (SARS-CoV-2). This led to increased clinical testing and decentralizing of this testing from provincial health laboratories to regional and private facilities. Leveraging the results from the Canadian Laboratory Response Network’s National SARS-CoV-2 Proficiency Test (PT) Program, this study compares multiple commercial and laboratory-developed nucleic acid amplification tests, assessing both sensitivity and specificity across multiple users.

**Methods:**

Each panel consisted of six blinded, contrived-clinical samples. Panels were distributed to international, provincial and territorial laboratories and subsequently to partner facilities. Participating laboratories were asked to run these sample through their respective extraction/PCR workflows and submit results to the National Microbiology Laboratory, outlining the nucleic acid extraction platform and nucleic acid amplification test employed, as well as the viral gene target and Ct values or equivalent obtained. Data were compiled for each molecular platform and gene target used.

**Results:**

The PT schemes were deployed in May 2020, November 2020 and June 2021, resulting in 683 data sets using 37 different nucleic acid amplification tests. Over the course of three PT schemes, the average score obtained was 99.3% by participants demonstrating consistent testing between laboratories and testing platforms.

**Conclusion:**

This study confirmed the rapid and successful implementation of a Canadian PT Program and provided comparative analysis of the various emergency use authorized and laboratory developed tests employed for the detection of SARS-CoV-2 and demonstrated an overall 99.3% test concordance nationwide.

## Introduction

In late 2019, a novel respiratory virus, severe acute respiratory coronavirus 2 (SARS-CoV-2), emerged in the Hubei province of China and subsequently caused the coronavirus disease 2019 (COVID-19) global pandemic. As the case numbers rapidly grew, it became necessary to decentralize testing to support testing at the federal, provincial/territorial and municipal levels, including private laboratories, hospitals and healthcare facilities.

The Canadian Laboratory Response Network (CLRN) at the National Microbiology Laboratory (NML) in Winnipeg, Canada provides high-consequence proficiency panels for biothreat agents to ensure that public health laboratories are ready to respond with high quality diagnostic testing. During the COVID-19 pandemic, the CLRN was leveraged to develop a Proficiency Test (PT) program to support facilities conducting SARS-CoV-2 clinical testing using molecular methods. Similar to other international efforts, the National SARS-CoV-2 PT Program supports the ability of public health testing facilities to establish competency and obtain or maintain accreditation to conduct SARS-CoV-2 clinical testing against a known reference standard to ensure consistency between testing platforms and laboratories across the country and across the globe ((1–3)). Nucleic acid amplification tests (NAAT) have been considered the gold standard method for the detection of active SARS-CoV-2 cases. Since the emergence of SARS-CoV-2 in December 2019, there have been a variety of NAATs developed, both laboratory-developed tests and commercial assays. This study provides a comparison of the various NAAT platforms employed within Canada over the course of three PT schemes from May 2020 to June 2021.

## Materials and methods

### Production, quality control and panel distribution

Irradiated viruses were diluted in a pooled, negative human nasal secretion as the background matrix at varying concentrations and immediately aliquoted into pre-labelled tubes. Each panel consisted of six blinded, contrived-clinical samples. Samples were sorted by site number, packaged appropriately for transport and stored at −80°C until distribution.

Prior to distribution, quality control measures were taken to ensure sample homogeneity and stability. In short, ten aliquots of each sample were removed from storage, nucleic acids were extracted as per manufacturer’s instructions (MagMax^TM^ CORE Nucleic Acid Purification Kit, Applied Biosystems^TM^, Ontario) and assayed by quantitative real-time polymerase chain reaction (qRT-PCR) (QuantiNova^®^ Probe RT-PCR Kit, Qiagen^®^, Ontario) targeting the E gene of SARS-CoV-2 ((4)). Coefficient of variations were calculated for each set of panel samples using GraphPad^®^ Prism’s descriptive statistics. An average Ct value with a coefficient of variation less than 10% was necessary to pass sample homogeneity quality controls. Stability testing began day 1 post-production and continued at specified intervals for the duration of the PT scheme using the same approach outlined above. If quality controls passed for homogeneity and stability testing on day 1 and seven post-production, the panels were released for distribution. Stability testing continued for the duration of the test scheme.

Panels were packed on dry ice and distributed to the international, provincial and territorial laboratories, who subsequently distributed panels to their partner facilities within their jurisdiction. Cold chain was monitored and if not maintained, a new panel was shipped directly from NML.

### Participant selection and intended use

Provincial and territorial members of the Canadian Public Health Laboratory Network (CPHLN) approached the NML to assist the pandemic response by producing and administering a SARS-CoV-2 PT Program, as one was not readily available at the time. The CPHLN provincial and territorial partners provided NML with a list of participants and were responsible for distribution of the test panels within their respective jurisdictions. Participants included provincial and territorial laboratories, public health laboratories, hospitals and healthcare facilities in both urban and rural communities. Specific metadata and details on individual site licensing and accreditation for SARS-CoV-2 were not made available to NML.

The PT panel was intended to be used as an internal validation of SARS-CoV-2 molecular processes, which are performed in conjunction with a nucleic acid extraction method. This panel was not intended to be used on platforms requiring fresh swab material, or the detection of viral antigens or virus-specific antibodies.

### Test result submission and analysis

Participating laboratories submitted results to NML outlining the nucleic acid extraction platform and NAAT employed, as well as the viral gene target and Ct values or equivalent obtained. Data were compiled for each molecular platform and gene target used. Coefficient of variation for each gene target within a single platform was determined using GraphPad^®^ Prism’s descriptive statistics. Probit analysis using a 95% cut-off was used to determine limit of detection based on sample detection ((5)).

## Results and discussion

The PT schemes were deployed in May 2020, November 2020 and June 2021, resulting in 683 data sets using 37 different NAAT (**Table 1**). Each PT scheme assessed assay sensitivity and specificity. The most commonly used platforms were fully automated low-throughput assays such as the DiaSorin Simplexa^TM^ COVID-19 Direct Molecular Assay, Cepheid Xpert^®^ Xpress SARS-CoV-2, Cepheid Xpert^®^ Xpress SARS-CoV-2/Flu/RSV and BioFire^®^ FilmArray RP2.1 Test Panel. These systems were employed mainly in hospital laboratories and in rural communities. Larger diagnostic centres, such as provincial laboratories and reference centres, generally employed high-throughput assays, including the Roche Cobas^®^ SARS-CoV-2 Test (for Cobas 6800/8800), Seegene Allplex^TM^ 2019 nCoV Assay, Thermo Fisher TaqPath^TM^ COVID-19 Combo Kit and LDT targeting the E gene (Table 1).

**Table 1:** Nucleic acid amplification test platforms utilized for the detection of SARS-CoV-2 during the Canadian Laboratory Response Network’s SARS-CoV-2 Proficiency Test Panels, May 2020 to June 2021

Nucleic acid amplification test platform		Proficiency test scheme, Number of sites/platform		
Manufacturer	Product name	May 2020	Nov 2020	June 2021
Abbott^TM^	Alinity^TM^ m SARS-CoV-2 AMP Kit	0	5	16
	SARS-CoV-2 Real Time PCR	1	3	3
Agena Bioscience	MassARRAY^®^ SARS-CoV-2 Panel	0	0	1
Altona	AltoStar^®^ SARS-CoV-2 RT-PCR Kit 1.5	1	1	2
BD	SARS-CoV-2 Reagents for the BD MAX^TM^ System	2	9	4
BGI^TM^	Real Time Fluorescent RT-PCR Kit for detecting SARS-CoV-2	0	2	1
BioFire^®^	Film Array^®^ Respiratory 2.1 Panel	0	20	49
Biomeme	SARS-CoV-2 Go Strips^TM^	0	1	1
Cepheid	Xpert^®^ Xpress SARS-CoV-2	34	36	52
	Xpert^®^ Xpress SARS-CoV-2/Flu/RSV	0	0	29
DiaSorin	Simplexa^TM^ COVID-19 Direct Molecular Assay	5	42	81
Hologic	Panther Fusion^®^ SARS-CoV-2 Assay	0	2	2
	Aptima^®^ SARS-CoV-2 Assay (Panther System)	0	6	8
Hyris	Virus Finder COVID-19 bKit^TM^	0	0	1
Laboratory-developed test	3’ UTR Target	0	0	1
	5’ UTR Target	0	2	4
	CDC CoVPlex Real-Time PCR Assay	0	0	1
	E Gene Target	12	27	49
	N Gene Target	1	1	10
	ORF1a/b Gene Target (RdRp)	5	5	8
	S Gene Target	0	1	0
	E and N Gene Pooled Targets	0	1	6
	E and ORF1a/b Gene Pooled Targets	0	0	1
	N, ORF1a/b and S Gene Pooled Targets	0	1	1
Luminex	Aries^®^ SARS-CoV-2 Assay	0	1	1
	NxTAG^®^ Respiratory Pathogen Panel + SARS-CoV-2	0	1	1
LuminUltra	GeneCount^®^ COVID-19 RT-qPCR Assay	0	0	1
Quidel	Lyra^®^ SARS-CoV-2 Assay	0	0	3
	Solana^®^ SARS-CoV-2 Assay	0	0	1
RIDA^®^ Gene	SARS-CoV-2 Test	0	2	1
Roche	Cobas^®^ SARS-CoV-2 Test (for Cobas 6800/8800)	13	6	19
	Cobas^®^ SARS-CoV-2 & Influenza A/B Test (for Cobas 6800/8800)	0	0	1
	Cobas^®^ SARS-CoV-2 (for Liat^®^)	0	0	1
	Cobas^®^ SARS-CoV-2 & Influenza A/B Assay (for Liat)	0	0	9
Seegene	Allplex^TM^ 2019 nCoV Assay	4	19	19
	Allplex^TM^ SARS-CoV-2/FluA/FluB/RSV Assay	0	0	1
Thermo Fisher Scientific	TaqPath^TM^ COVID-19 Combo Kit	1	6	15
Total number of results submitted		79	200	404

Abbreviations: CDC, Centers for Disease Control and Prevention; COVID-19, coronavirus disease 2019; PCR, polymerase chain reaction; RT-PCR, real-time polymerase chain reaction; RSV, respiratory syncycial virus; SARS-CoV-2, severe acute respiratory syndrome coronavirus 2

**Figure 1:**
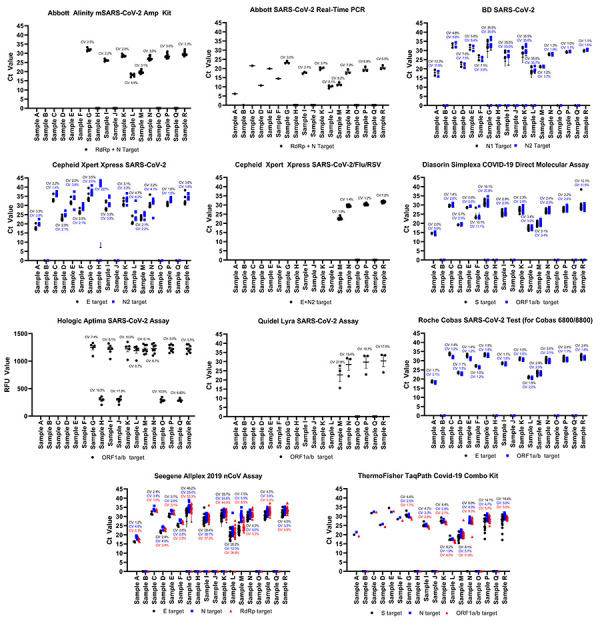
Commercial nucleic acid amplification test performance obtained during the Canadian Laboratory Response Network’s SARS-CoV-2 Proficiency Test Program, May 2020 to June 2021^a^ Abbreviations: COVID-19, coronavirus disease 2019; PCR, polymerase chain reaction; RFU, relative fluorescence unit; RSV, respiratory syncycial virus; SARS-CoV-2, severe acute respiratory syndrome coronavirus 2 ^a^ Ct values are presented for each nucleic acid amplification platform tested. Each data point is presented with the mean and standard error. The coefficient of variation is denoted for each target in its respective colour. Data points at the 0 value on the axis indicate there were no detectable SARS-CoV-2 RNA

Panel results obtained using commercially available NAATs that have at least three datasets in any given test scheme are presented in **Figure 1**. Infrequently used platforms were not assessed further. Abbott produces two high-throughput, laboratory-based molecular assays for the detection of SARS-CoV-2: the Alinity m SARS-CoV-2 AMP Kit used with the Alinity m System; and SARS-CoV-2 RealTime PCR employing the m2000 RealTime System. Both systems obtained expected results for all samples across three test schemes. All sites demonstrated consistent results from November 2021 to June 2021 with coefficient of variations less than 10% (Figure1).

The BD SARS-CoV-2 Reagents for the BD MAX System targeting the N gene were utilized for the detection of SARS-CoV-2. The BD MAX System is a fully automated system, allowing the user to run up to 24 samples at a time. Over the course of 13 months, the BD SARS-CoV-2 Reagents for the B MAX System performed with variable accuracy. During the May 2020 test scheme, samples were accurately detected in all cases, but the coefficient of variation ranged from 4.8%–12.3%, indicating increased variation between users. Discordant results were observed during the November 2021 test scheme; 6/7 failures to detect SARS-CoV-2 were attributed to user error (Figure 1, **Table 2**); therefore, the data obtained for Sample G–L were skewed and the accuracy and consistency were negatively affected. Removing these data points would regain an overall 100% target accuracy for the N1 target and 99% accuracy for N2; the latter target failed to identify the presence of Sample I (Figure 1, Table 2). During the June 2021 test scheme, the BD SARS-CoV-2 Reagents for the BD MAX System performed with 100% accuracy. Ct values were consistent among all users denoted by a coefficient of variations of less than 5% (Figure 1).

**Table 2:** Nucleic acid amplification test platform discordant target results for SARS-CoV-2 obtained with the Canadian Laboratory Response Network’s SARS-CoV-2 Proficiency Test Panels, May 2020 to June 2021

PCR platform	Assay target	PCR platform SARS-CoV-2 discordant results (%)	Sensitivity: 95% detection^a,b^ (copies/ml)	Specificity	
				Positive agreement (%)^b^	Negative agreement (%)^b^
BD SARS-CoV-2 Reagents for the BD MAX^TM^ System	N1	6/96 (6.25%)	1,100 or fewer	100	100
	N2	7/96 (7.29%)	1,100 or fewer	100	100
BioFire^®^ Film Array^®^ RP2.1	M/S	1/414 (0.24%)	1,100 or fewer	100	100
Cepheid Xpert^®^ Xpress SARS-CoV-2	E	0/730 (0.00%)	1,100 or fewer	100	100
	N	6/730 (0.82%)	1,100 or fewer	100	97
DiaSorin Simplexa^TM^ COVID-19 Direct Molecular Assay	ORF1a/b	3/768 (0.39%)	1,100 or fewer	99.7	100
	S	4/768 (0.52%)	1,100 or fewer	99.6	100
NxTAG^®^ Respiratory Pathogen Panel + SARS-CoV-2	ORF1a/b	0/12 (0.00%)	1,100 or fewer	100	100
	M	1/12 (8.33%)	1,100 or fewer	100	100
RIDA^®^ gene SARS-CoV-2 Test	E	2/18 (11.11%)	1,100 or fewer	100	100
Seegene Allplex^TM^ 2019 nCoV Assay	E	11/252 (4.37%)	1,100 or fewer	100	100
	RdRp	14/252 (5.56%)	1,358	99.1	100
	N	9/252 (3.57%)	1,100 or fewer	100	100

Abbreviations: PCR, polymerase chain reaction; SARS-CoV-2, severe acute respiratory syndrome coronavirus 2

^a^ Assessment using Sample C, G and P

^b^ Off-label procedural methods and user errors were removed from the assessment

The BioFire Film Array RP2.1 test kit uses a fully automated system to test for the presence of 22 different pathogens, including SARS-CoV-2. This assay has a nucleic acid extraction step followed by reverse transcription/nested PCR step coupled with deoxyribonucleic acid melt curve technology to identify the presence of target pathogens qualitatively. Out of 414 samples tested, it missed identifying the presence of SARS-CoV-2 in only one sample; demonstrating a 99.8 concordance rate (**Table 3**). One site was unable to detect SARS-CoV-2 in Sample P; however, it was determined that insufficient mixing of the test sample was likely responsible for the discrepant results. Furthermore, this site correctly identified the presence of other target pathogens, which were present in the samples such as rhinovirus (Sample M), respiratory syncytial virus (Sample K), influenza A virus (Sample H and O) and influenza B virus (Sample R) (Table 3).

**Table 3:** Qualitative performance of the BioFire Film Array Respiratory 2.1 Panel and Roche Cobas SARS-CoV-2 and Influenza A/B Assay (for Liat) during the Canadian Laboratory Network’s SARS-CoV-2 proficiency test schemes, May 2020 to June 2021

Platform		Sample ID	Sample G	Sample H	Sample I	Sample J	Sample K	Sample L
BioFire^®^ Film Array^®^ Repiratory Panel 2.1	Expected results		Detected SARS-CoV-2	Detected Influenza A	Detected SARS-CoV-2	No agent detected	Detected SARS-CoV-2 RSV	Detected SARS-CoV-2
	Sample concordance		100% (20/20)	100% (20/20)	100% (20/20)	100% (20/20)	100% (20/20)	100% (20/20)
	Sample ID		Sample M	Sample N	Sample O	Sample P	Sample Q	Sample R
	Expected results		Detected SARS-CoV-2 rhinovirus	Detected SARS-CoV-2	Detected Influenza A	Detected SARS-CoV-2	No agent detected	Detected SARS-CoV-2 Influenza B
	Sample concordance		100% (n=49/49)	100% (n=49/49)	100% (n=49/49)	98.6% (n=48/49)	100% (n=49/49)	100% (n=49/49)
	Overall concordance		99.8% (413/414)					
Roche Cobas^®^ SARS-CoV-2 & Influenza A/B Assay (for Liat^®^)	Sample ID		Sample M	Sample N	Sample O	Sample P	Sample Q	Sample R
	Expected results		Detected SARS-CoV-2	Detected SARS-CoV-2	Detected Influenza A	Detected SARS-CoV-2	No agent detected	Detected SARS-CoV-2 Influenza B
	Sample concordance		100% (n=9/9)	100% (n=9/9)	100% (n=9/9)	100% (n=9/9)	100% (n=9/9)	100% (n=9/9)
	Overall concordance		100% (n=54/54)					

Abbreviations: RSV, respiratory syncycial virus; SARS-CoV-2, severe acute respiratory syndrome coronavirus 2

The Cepheid GeneXpert platform is readily used across Canada for the detection of SARS-CoV-2 employing the Xpert Xpress SARS-CoV-2 and Xpert Xpress SARS-CoV-2/Flu/RSV assays. The Xpert Xpress SARS-CoV-2 E assay performed with accuracy (100% detection rate) and consistency (coefficient of variation less than 5%) for all samples; however, discordant results were observed using the N target, specifically for Sample H. Sample H did not contain SARS-CoV-2 but did contain a moderate amount of influenza A virions (Ct 27); there were six instances where the SARS-CoV-2 N2 target produced a Ct greater than 40, which was deemed positive for SARS-CoV-2 by the GeneXpert software (Figure 1, Table 2). Apart from Sample H, the Ct values for the N target were consistent and had a coefficient of variation less than 10%, Figure 1. The recently developed Cepheid Xpert Xpress SARS-CoV-2/Flu/RSV assay was employed during the June 2021 test scheme and the result output for SARS-CoV-2 was combined for both E and N2 targets. The platform had a 100% accuracy and produced very consistent results with a coefficient of variation less than 2% among all users (Figure 1). The Xpert Xpress SARS-CoV-2/Flu/RSV assay also correctly identified the presence of influenza A and B in Samples O and R, respectively (data not shown).

The Diasorin Simplexa COVID-19 Direct Molecular Assay is a low throughput, automated system that can run up to eight samples at once. Its main distinction from other similar systems, such as the BioFire Film Array and Cepheid GeneXpert platforms, is that it eliminates the nucleic acid extraction/purification step. Discordant results were observed for Sample G and Sample R, the ORF1a/b target missed detecting SARS-CoV-2 n=2/768 times (0.26%), while the S target did not detect SARS-CoV-2 n=3/768 times (0.39%) (Figure 1, Table 2). According to the manufacturer, the S assay has a 95.8% detection rate of 500 copies/ml (2,000 copies/ml for 100% detection) and the ORF1a/b is detected 93.8% of the time at 1,000 copies/ml (2,000 copies/ml for 100% detection ((6)). Similar observations were observed here: the S assay performed better than the ORF1a/b assay (Table 2). Sample G and R are approximately 1,100 and 3,500 copies/ml respectively, which is the range of the assay’s limit of detection (LOD) for both targets, and is the likely cause for the discrepant results (**Table 4**). Furthermore, there was an additional discordant result for each target due to a software error that reported “no result” when Ct values were obtained for both targets (Table 2). For samples where all targets were correctly identified (Samples A–F and H–Q), coefficient of variations were 5% or less, except for Sample F which had coefficients of variations of 11.1% and 10.1% for the ORF1a/b and S targets, respectively (Figure 1).

**Table 4:** Sample identity and approximate viral loads for test samples provided during the Canadian Laboratory Network’s SARS-CoV-2 proficiency test schemes, May 2020 to June 2021

Sample	Identity	SARS-CoV-2 E approximate copies/ml	Approximate Ct value (SARS-CoV-2 E target)^a^
**CLRN’s SARS-CoV-2 proficiency test scheme – May 2020**			
A	SARS-CoV-2 wild type	120,000,000	20
B	Blank	0	0
C	SARS-CoV-2 wild type	1,600	36
D	SARS-CoV-2 wild type	2,700,000	25
E	SARS-CoV-2 wild type	3,900	35
F	SARS-CoV-2 wild type	216,000	29
**CLRN’s SARS-CoV-2 proficiency test scheme – November 2020**			
G	SARS-CoV-2 wild type	1,100	36
H	Influenza A virus	0	0
I	SARS-CoV-2 wild type	54,000	31
J	Blank	0	0
K	SARS-CoV-2 wild type	10,800	33
	Respiratory syncytial virus	0	
L	SARS-CoV-2 wild type	13,000,000	22
**CLRN’s SARS-CoV-2 proficiency test scheme – June 2021**			
M	SARS-CoV-2 B.1.351	280,000	28
	Rhinovirus	0	
N	SARS-CoV-2 B.1.1.7	2,100	35
O	Influenza A virus	0	0
P	SARS-CoV-2 P.1	1,600	36
Q	Blank	0	0
R	SARS-CoV-2 wild type	3,500	35
	Influenza B virus	0	

Abbreviations: CLRN, Canadian Laboratory Response Network; SARS-CoV-2, severe acute respiratory syndrome coronavirus 2

^a^ Corman *et al.* reference ((4))

Hologic produces two SARS-CoV-2 assays that were employed during the scope of the CLRN SARS-CoV-2 PT schemes: Panther Fusion SARS-CoV-2 assay and Aptima SARS-CoV-2 assay. The Panther Fusion SARS-CoV-2 assay was not presented here as only two sites employing this platform, while the Aptima SARS-CoV-2 assay was employed during the November 2020 and June 2021 test schemes with six and eight users respectively (Table 1). This platform demonstrated 100% concordance (n=90/90 samples); however, the Ct values obtained were quite variable, with coefficients of variation ranging from 5% to 19.5% across samples (Table 1).

During the June 2021 CLRN PT scheme, the Quidel Lyra SARS-CoV-2 Assay targeting the ORF1a/b was employed for the first time by three participants (Table 1). This assay was able to correctly identify all test samples (n=18); however, the variability between Ct values was large, with a coefficient of variations ranging from 17.9 to 27.8 (Figure 1). This variation in Ct values is largely attributed to one set of test panel results, which provided substantially lower Ct values than the other participants, indicating differences in threshold settings between participants.

The Seegene Allplex 2019 nCoV Assay is a multiplex RT-PCR assay that detects the E, N and RdRp targets and can be automated for high volume testing. This test performed well during the May 2020 and June 2021 PT schemes demonstrating a 100% concordance and consistent results conveyed by a coefficient of variation less than 10% (Figure 1); however, a number of discordant results were observed during the November 2020 PT scheme, causing subsequent decreases in reproducibility and elevated coefficients of variation. Sample G was associated with n=3/19 E target failures, n=4/19 RdRp target failures and n=1/19 N target failures. While n=2/19 RdRp target failures were associated with the use of a nucleic extraction platform, the remaining failures were associated with a divergence from manufacturer’s recommendations and did not employ a nucleic acid extraction step. Furthermore, the reported LOD for the Seegene Allplex 2019 nCoV Assay is approximately 4,000 copies/ml, which is higher than the Sample G titer and is likely responsible for the failure to detect SARS-CoV-2 in this sample ((7)) (Table 4) Conversely, Sample I was associated with n=1/19 E target failures and n=2/19 RdRp and N target failures; while Sample K had n=2/19 E target failures, n=3/19 RdRp target failures and n=1/19 N target failures. Sample L, H and J were also associated with one discordant result for each target due to the inability to acquire a valid result. These remaining failures to detect SARS-CoV-2 were all associated with off-label use of not employing a nucleic acid extraction procedure, and are likely the cause of the discordant result since sample titers were all above 4,000 copies/ml. The practice of not implementing an extraction protocol was not observed in the subsequent test scheme. Overall, the E, RdRp and N targets produced discordances of 4.37%, 5.56% and 3.52%, respectively (Figure 1, Table 2).

Two different Roche assays were utilized during the CLRN SARS-CoV-2 PT schemes, Roche Cobas SARS-CoV-2 Test, a fully automated, high throughput assay intended for use with the Roche Cobas 5800/6800/8800, and Roche Cobas SARS-CoV-2 & Influenza A/B Assay for Liat, a fully automated qualitative point of care test to be used on the Cobas Liat. The Roche Cobas SARS-CoV-2 Test for Cobas 5800/6800/8800 was employed during all three test schemes, producing accurate and consistent results with the coefficient of variations less than 3% (Figure 1). The Roche Cobas SARS-CoV-2 Test for Liat accurately detected all test samples from nine users (Table 1 and Table 3). Overall, the Roche Cobas SARS-CoV-2 Test for use on the Cobas 5800/6800/8800 performed the best when comparing commercial platforms across the CLRN SARS-CoV-2 PT schemes; it demonstrated 100% accuracy and produced the most reproducible results across users.

**Figure 2:**
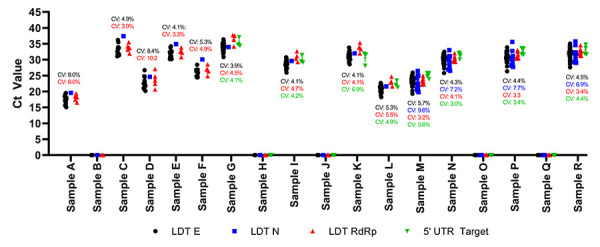
Laboratory-developed nucleic acid amplification test performance obtained during the Canadian Laboratory Response Network SARS-CoV-2 Proficiency Test Program, May 2020 to June 2021^a^ Abbreviations: LTD, laboratory-developed tests; SARS-CoV-2, severe acute respiratory syndrome coronavirus 2 ^a^ Ct values are presented for each nucleic acid amplification platform tested. Each data point is presented with the mean and standard error. The coefficient of variation is denoted for each target in its respective colour. Data points at the 0 value on the axis indicate there was no detectable SARS-CoV-2 RNA

The LDT were also employed during the CLRN SARS-CoV-2 PT Scheme from May 2020 to June 2021. Data sets obtained using LDTs that have at least three sets of submitted results in any given test scheme are presented (**Figure 2**). In all cases, all tests were able to detect SARS-CoV-2 effectively and accurately from the test samples provided (Figure 2). The E and RdRp targets were used in all test schemes (Table 1). The reproducibility of the E target and RdRp target ranged from coefficients of variation between 3.9% and 8.4% and between 3.2% and 10.2%, respectively (Figure 2). The use of the 5’ UTR target emerged during the November 2020 test scheme and results were consistently detected with coefficients of variation less than 7% (Figure 2). Laboratories began employing the N target test during the June 2021 test scheme with coefficients of variation ranging between 6.9% and 9.6% (Figure 2). It should be noted that, apart from the targeted gene, we do not have the specific details regarding the primer/probe sequences implemented by each user and it is possible that the sequences utilized are different. In general, Ct values were similar between all the target tests indicating similar detection affinities; however, a more detailed direct comparative analysis was not conducted, since the assays were not identical. Furthermore, shifts between gene targets are expected, as individual gene expression may differ during viral replication; but this finding could also be attributed to technical variations in the threshold/detection settings by different laboratories. Overall, the 5’ UTR target on average demonstrated the most consistent results with an average coefficient of variation of 4.3%, followed by the RdRp (4.7%), E (5.2%) and N (7.9%) targets. All targets performed within designated specifications of coefficients of variation of less than 10%.

Overall, these results provide insights into test sensitivity; each test scheme involved testing a sample, which contained low concentrations of virus particles, ranging from 1,100 to 1,600 copies/ml (Sample C, 1,600 copies/ml, Sample G, 1,100 copies/ml or Sample P, 1,600 copies/ml). Effective test sensitivity was observed across all presented commercial and LDT assays employed across the country. A 100% concordance rate for these low concentration samples was observed for all SARS-CoV-2 targets, with a few exceptions. The BioFire Film Array RP2.1 test kit missed detecting Sample G 1/414 times (Table 3); however, this error occurred due to a procedural mishandling of the sample, and upon repetition for remediation purposes, it was detected. Therefore, this error was not included in the general assessment of sensitivity (Table 3).

The Diasorin Simplexa COVID-19 Direct Molecular Assay missed detecting two low concentration samples, both targets were unable to detect Sample G on two occurrences and the S target failed to detect Sample R (3,500 copies/ml) in one instant (Table 2); however, these discordant results did not cause the 95% limit of detection rate to be affected. The Seegene Allplex 2019 nCoV Assay was associated with a number of failures to detect Sample G. The majority of these failures were attributed to off-label use, where a required nucleic acid extraction process was omitted; for this reason, these results were removed from the subsequent analysis of sensitivity. However, there were two instances associated with proper use, where the RdRp target failed to identify SARS-CoV-2 and were included in the analysis. These discordant results elicited a minor effect on test sensitivity; a 95% detection limit was determined to be 1,358 copies/ml (Table 2). With the exception of the Seegene Allplex 2019 nCoV assay, all other assays had 95% detection limits below 1,100 copies/ml. These observed results are in line with the manufacturers reported limits of detection for their respective assays ((6–16)). While outside of the scope of the intended use of this PT scheme, this study was not able to calculate the limit of detection for all the assays due to lack of samples below detectable levels and therefore further comparison of assay sensitivity was not possible.

In addition to test sensitivity, specificity of the assays was also assessed during the PT schemes. More specifically, the May 2020 PT scheme focused on positive and negative agreement, while the November 2020 test scheme added a component for the detection of other respiratory pathogens of significance, and finally the June 2021 test scheme built upon the last by including relevant SARS-CoV-2 variants of concern (Table 4). Negative agreement for Sample B was 100% across all platforms. The November 2020 test scheme consisted of two samples, neither of which contained SARS-CoV-2: instead, Sample H contained a moderate dose of influenza A virus (Ct 27) and Sample J contained the negative nasal secretion/UTM matrix only. Sample J had 100% negative agreement across all platforms; however, Sample H demonstrated some inconsistencies when the Cepheid Xpert Xpress SARS-CoV-2 platform was employed. In six instances, according to the manufacturer’s instructions for reporting, the N target incorrectly identified the presence of SARS-CoV-2 in a sample that only contained influenza A virus (Table 2). In each circumstance, the Ct values were >40 and suggested that there was some degree of cross reactivity with influenza A virus, as this was never observed with any of the negative samples. Since, all discordant results were above the 40 Ct value, recommendations were made to investigate modifying the Ct cut-off to 40 instead of 45, as recommended by the manufacturer to avoid reporting false positives ((17)). Over the course of the three PT schemes, the Cepheid Xpert Xpress SARS-CoV-2 platform had a 100% negative agreement for the E target and a 97% negative agreement for the N target. Negative agreement for Sample O and Q were 100% across all platforms.

All commercial and laboratory developed tests were successfully able to detect the variants of concern. Of note, the Thermo Fisher TaqPath COVID-19 Combo Kit had a drop off in one of its three target genes; the S gene was not able to detect the B.1.1.7 variant, while the other two target genes were successfully identified. According to the manufacturer’s recommendations for reporting, a positive result requires n=2/3 targets to have Ct values less than 37; therefore, the loss of the S gene did not impair the assays ability to detect the presence of SARS-CoV-2 in Sample N ((14)). Failure of the BioFire Film Array RP2.1 to detect SARS-CoV-2 P.1 was attributed to a technical error and not an assay failure; therefore, this test was not included in the analysis. The BioFire Film Array RP2.1 successfully detected the P.1 variant in all other attempts (n=48).

Overall, test specificity was comparable across all three PT schemes and platforms; a 99.5% negative agreement was observed.

## Conclusion

Over the course of three PT schemes conducted across Canada between May 2020 and June 2021, the average score obtained by participants was 99.3%, demonstrating consistent testing between laboratories and testing platforms. Similarly high levels of agreement have been observed internationally. The American Proficiency Institute conducted a study across the United States and reported an overall score greater than 97% ((3)). Similarly, the Royal College of Pathologists of Australasia conducted three PT schemes within Australia and New Zealand between March 2020 and November 2020, with an initial score of 75% concordance early in the pandemic but then dramatically increasing to 95% concordance in the two latter test schemes ((2)). Finally, a third program from South Korea demonstrated 93% agreement ((1)). While each program varied in its sample composition and intended uses, it is encouraging to see that rapid deployment of SARS-CoV-2 testing resulted in consistently high degrees of agreement across the globe.

The ability to support quality assurance of testing measures through the provision of an external PT Program is essential during a novel or emerging public health threat. CLRN provides a framework to support the quality assurance required for the decentralization and increase in testing capacity within Canada. All Canadian public health laboratories follow a quality management program required by their respective jurisdictions, and on-site verification and validation schemes are essential to achieve these processes. Furthermore, the comparison of PT panel results allows for the assessment of various NAAT platforms at different locations across multiple users providing an overall assessment of platform performance. The cumulative performance of the NAAT employed during the three CLRN SARS-CoV-2 PT schemes was 99.3% concordant. A future consideration would be to collect additional data from participants to gain a greater scope of demographics, population statistics and accreditation status. This study demonstrates the rapid and successful implementation of a Canadian PT Program and provided comparative analysis of the various emergency use authorized and laboratory developed tests employed for the detection of SARS-CoV-2.
